# Supplementary material for: Spatial analysis of gut microbiome reveals a distinct ecological niche associated with the mucus layer
Source: Gut Microbes. 2021 Feb 11;13(1):1874815. doi: 10.1080/19490976.2021.1874815 (PMC8253138; doi:10.1080/19490976.2021.1874815)
Supplement: Supplemental Material [file KGMI_A_1874815_SM4091.zip › Supplementary information/Supplementary legends.docx]

**Supplementary Figure 1. Characterization of dense community structure**

1. Gel of PCR amplified samples extracted from 1^st^ 100-μm germ-free (GF) mice (lanes 1-3), 1^st^ 100-μm conventional mice (lanes 4-6), subsequent 50-μm GF mice (lanes 7-9), and subsequent 50-μm conventional mice (lanes 10-12) showing no PCR amplification from GF samples. Lane 13 negative control. Lane 14 positive control.
2. Class level relative abundance of inner and outer communities (N = 6 mice from Cage 1, SD).
3. Differential abundance of class Gammaproteobacteria calculated using DESeq2, showing significantly more Gammaproteobacteria in the outer community (N = 6 mice from Cage 1)
4. Observed ASVa showing higher alpha diversity in inner community.
5. Chao1 diversity index showing higher richness in the inner community (N = 6 mice from Cage 1).
6. Shannon diversity index showing no difference in Shannon diversity between inner and outer communities (N = 6 mice from Cage 1).
7. Pielou’s Evenness index showing significantly higher evenness in the outer community (N =6 mice from Cage 1).

**Supplementary Figure 2. Dense community and mucus thickness in colon of Jackson mice.**

1. FISH image identifying all bacteria (green) to show biofilm-like structure close to host epithelium (DAPI, blue).
2. Mean fluorescence intensity (MFI) measurements from a representative mouse to show bacterial density of biofilm-like structure (N = 3 mice, SEM).
3. Relative frequency of dense mucus layer thickness measurements, described in Methods section under “Mucus Thickness” (N = 5 mice, 40 measurements each).
4. Threshold cycle (Ct) values of 16s rRNA gene in inner and outer community.
5. Alpha rarefaction curves plotting the number of unique amplicon sequence variants (ASVs) from inner (red) and outer (blue) communities showing more unique ASVs are found in the inner community (N = 5 mice).
6. Observed ASVs showing significantly higher richness in the inner community (N = 5 mice).
7. Chao 1 diversity index showing significantly higher richness in the inner community (N = 5 mice).

**Supplementary Figure 3. Differences in fecal communities between WT and Rag1KO mice**

1. Principal coordinates of analysis (PcoA) plot using weighted UniFrac distances shows there is a significant difference between fecal communities of conventionalized WT and Rag1KO mice (N = 5 mice per group).
2. Principal coordinates of analysis (PcoA) plot using unweighted UniFrac distances shows there is no significant difference between fecal communities of conventionalized WT and Rag1KO mice (N = 5 mice per group).
3. Family level relative abundance of fecal communities in conventionalized Rag1KO and WT mice (N = 5 mice per group, SD)
4. Differential abundance of family Prevotellaceae calculated using DESeq2, showing significantly less Prevotellaceae in the fecal communities of Rag1KO mice (N = 5 mice per group)

**Supplementary Figure 4. Comparison between AL and IF Fecal communities**

1. Whole colonic cross section stained with fluorescent probes identifying all bacteria (green) and host epithelium (blue) of dense community structure in *Ad libitum* mice
2. Whole colonic cross section stained with fluorescent probes identifying all bacteria (green) and host epithelium (blue) of dense community structure in Intermittent fasting mice.
3. Observed ASVs found in AL and IF fecal communities showing no significant difference in richness between communities (N = 6 mice per group, SEM)
4. Shannon diversity in AL and IF fecal communities showing no significant difference in diversity between communities (N = 6 mice per group, SEM)
5. Principal coordinates of analysis (PCoA) plot using weighted UniFrac distances shows fecal communities from AL and IF are not significantly different (N = 6 mice per group)
6. Principal coordinates of analysis (PCoA) plot using unweighted UniFrac distances shows fecal communities from AL and IF are significantly different (N = 6 mice per group).
