# Supplementary figures and images for: Spatial analysis of gut microbiome reveals a distinct ecological niche associated with the mucus layer
Source: Gut Microbes. 2021 Feb 11;13(1):1874815. doi: 10.1080/19490976.2021.1874815 (PMC8253138; doi:10.1080/19490976.2021.1874815)

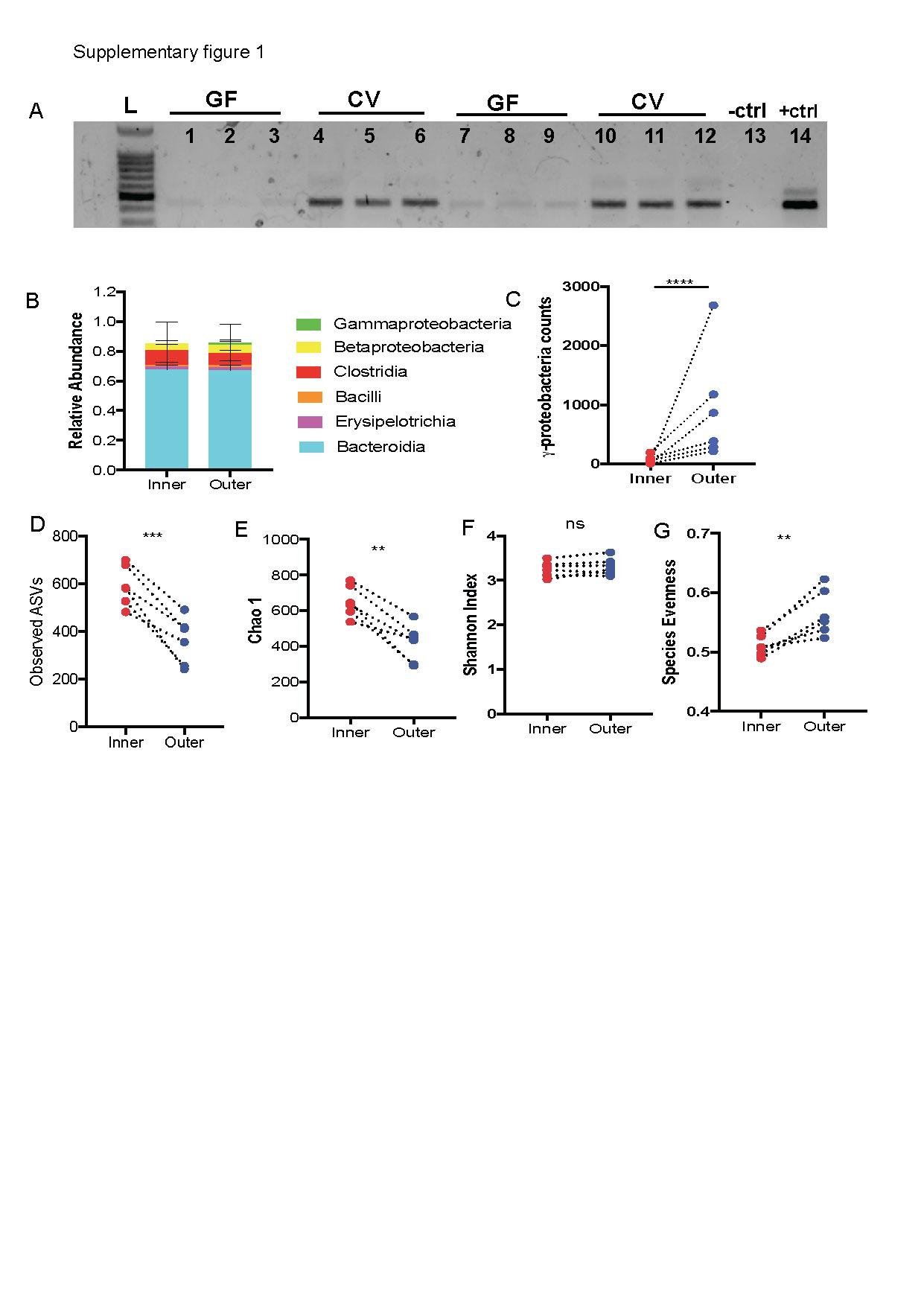

Supplement: Supplemental Material [file KGMI_A_1874815_SM4091.zip › Supplementary information/S1_01.jpg]

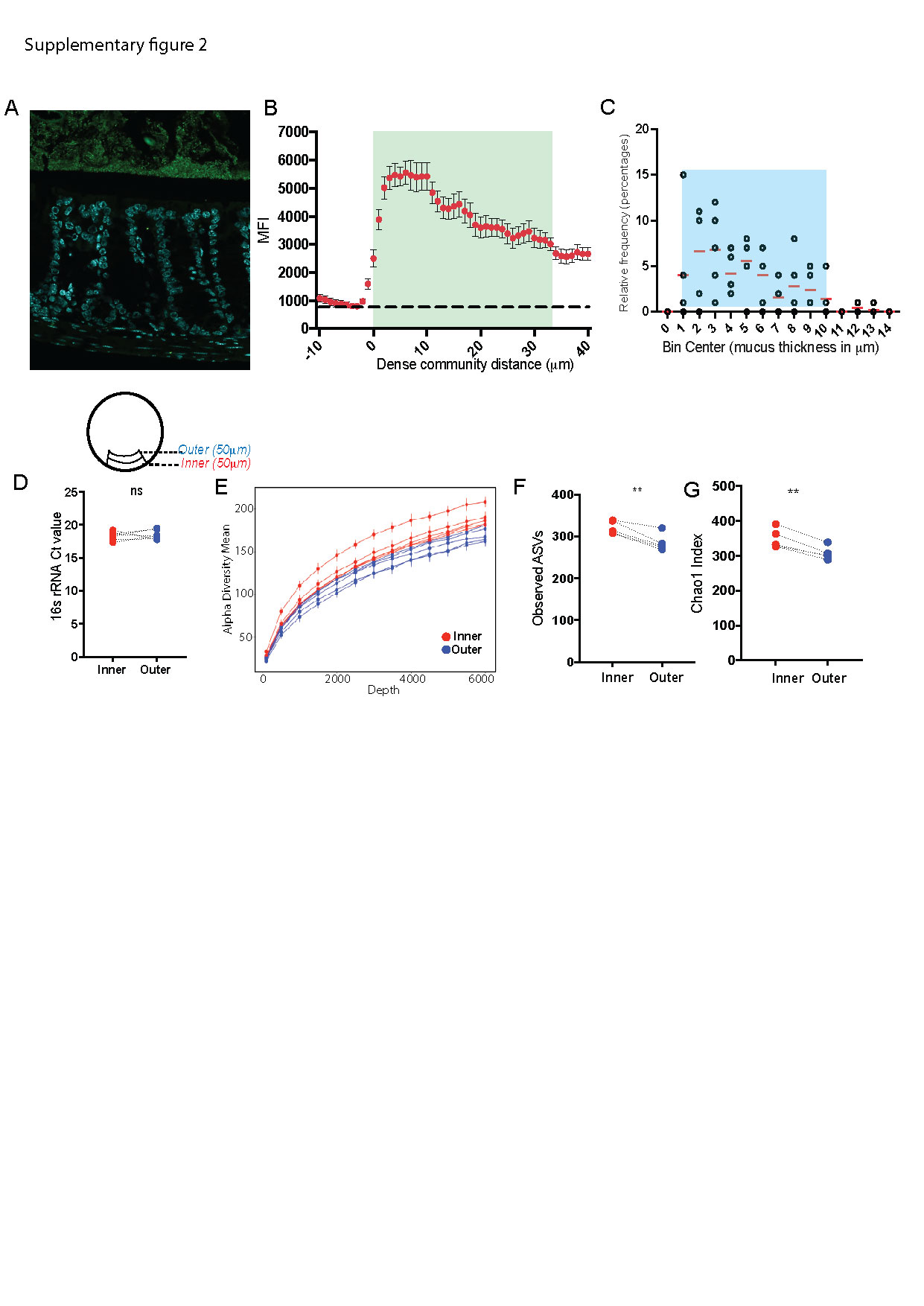

Supplement: Supplemental Material [file KGMI_A_1874815_SM4091.zip › Supplementary information/S2_01.jpg]

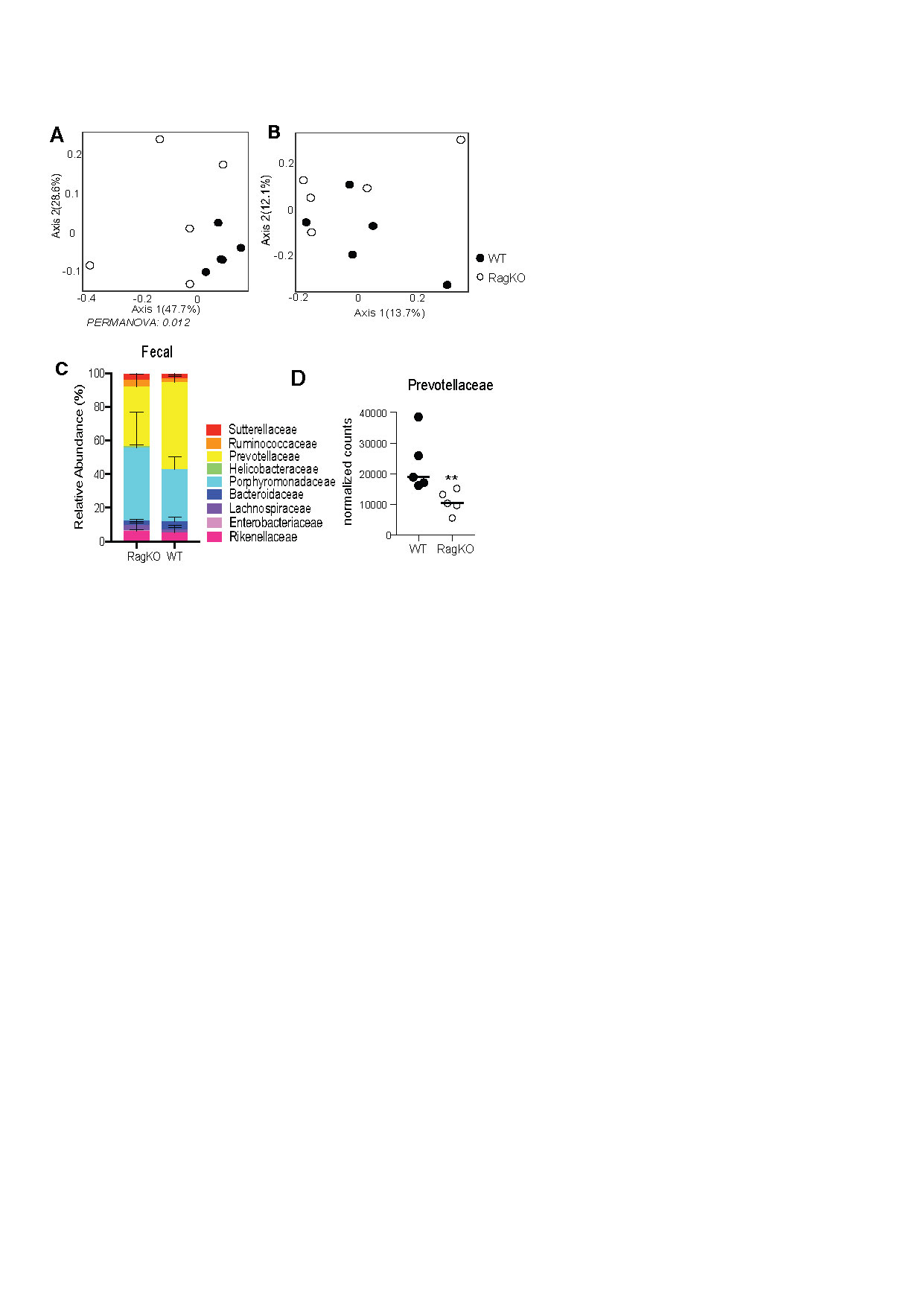

Supplement: Supplemental Material [file KGMI_A_1874815_SM4091.zip › Supplementary information/S3_01.jpg]

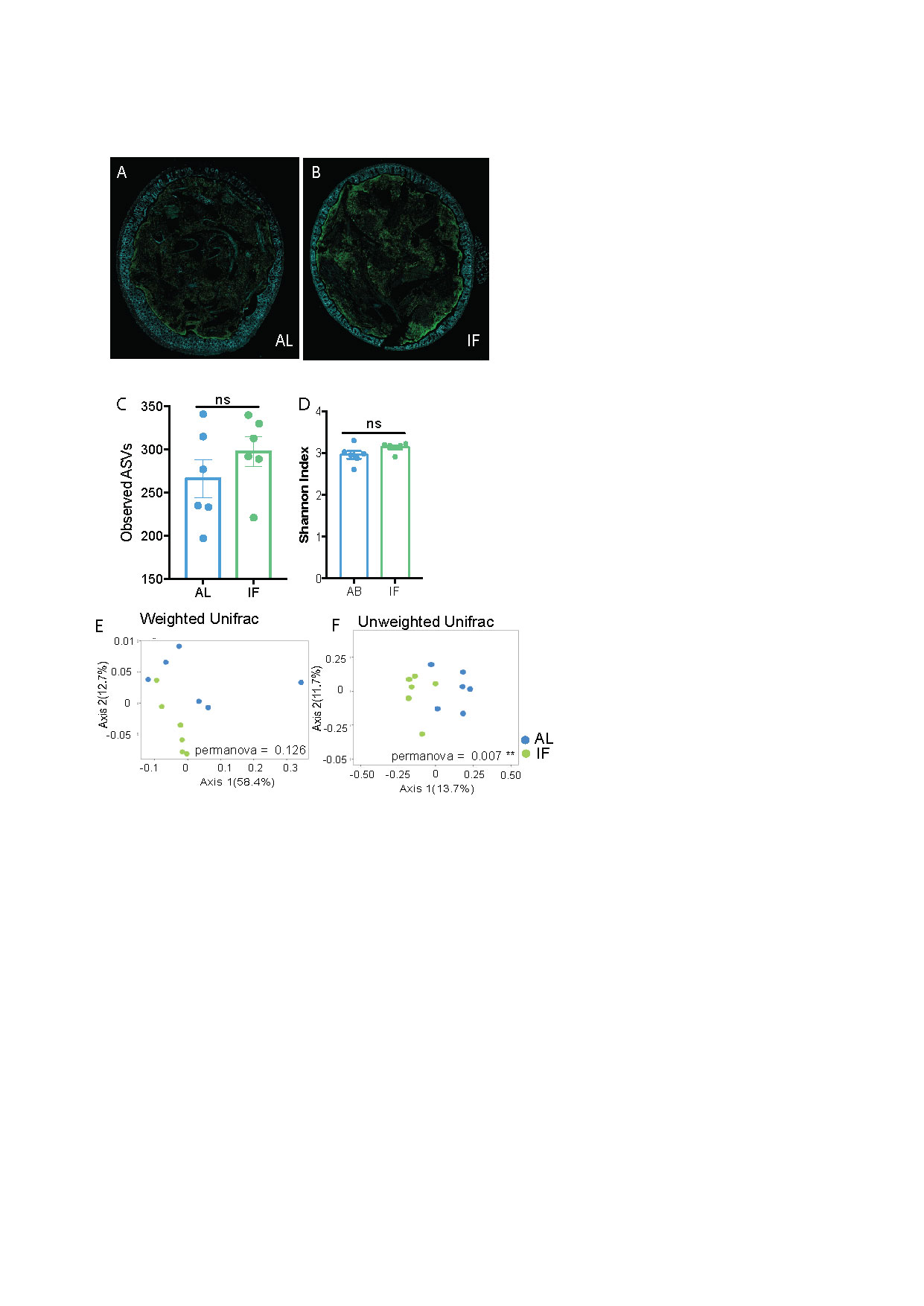

Supplement: Supplemental Material [file KGMI_A_1874815_SM4091.zip › Supplementary information/S4_01.jpg]
